# RNA transport during TMV cell-to-cell movement

**DOI:** 10.3389/fpls.2012.00193

**Published:** 2012-08-28

**Authors:** Eduardo J. Peña, Manfred Heinlein

**Affiliations:** ^1^Institut de Biologie Moléculaire des Plantes du Centre National de la Recherche Scientifique, Université de StrasbourgStrasbourg, France; ^2^Department of Plant Physiology, University of BaselBasel, Switzerland

**Keywords:** *Tobacco mosaic virus*, plasmodesmata, RNA transport, RNA labeling, endoplasmic reticulum, cytoskeleton, movement protein, replication complex

## Abstract

Studies during the last 25 years have provided increasing evidence for the ability of plants to support the cell-to-cell and systemic transport of RNA molecules and that this process plays a role in plant development and in the systemic orchestration of cellular responses against pathogens and other environmental challenges. Since RNA viruses exploit the cellular RNA transport machineries for spreading their genomes between cells they represent convenient models to investigate the underlying mechanisms. In this regard, the intercellular spread of *Tobacco mosaic virus* (TMV) has been studied for many years. The RNA of TMV moves cell-to-cell in a non-encapsidated form in a process depending on virus-encoded movement protein (MP). Here, we discuss the current state of the art in studies using TMV and its MP as a model for RNA transport. While the ability of plants to transport viral and cellular RNA molecules is consistent with RNA transport phenomena in other systems, further studies are needed to increase our ability to visualize viral RNA (vRNA) *in vivo* and to distinguish RNA-transport related processes from those involved in antiviral defense.

*Tobacco mosaic virus* (TMV) is a rod-shaped virus with a positive-sensed RNA genome that encodes 126 kD and 183 kD subunits of replicase, a 30 kD movement protein (MP) and a 17.5 kD coat protein (CP). The virion consists of the viral RNA (vRNA) protected by 2130 subunits of assembled CP. The virus has been a paradigm for RNA virus movement since the requirement of its MP for virus movement was defined (Deom et al., 1987; Citovsky, 1999; Waigmann et al., 2007). In addition, the virus provides a valuable tool to study RNA transport as the spread of infection does not require CP and the vRNA moves cell-to-cell in a non-encapsidated form. The MP has sequence-nonspecific binding affinity to single-stranded nucleic acids *in vitro* (Citovsky et al., 1990) and likely forms a ribonucleoprotein (vRNP) complex with vRNA upon replication *in vivo*. Based on experimental evidence suggesting that also the replicase is involved in virus movement (Hirashima and Watanabe, 2001; Guenoune-Gelbart et al., 2008) and that the virus moves in the form of replication complexes (Kawakami et al., 2004), the vRNP may be associated with several viral and host proteins in addition to vRNA and MP.

## vRNA transport occurs via the ER/actin network

**Figure 1:**
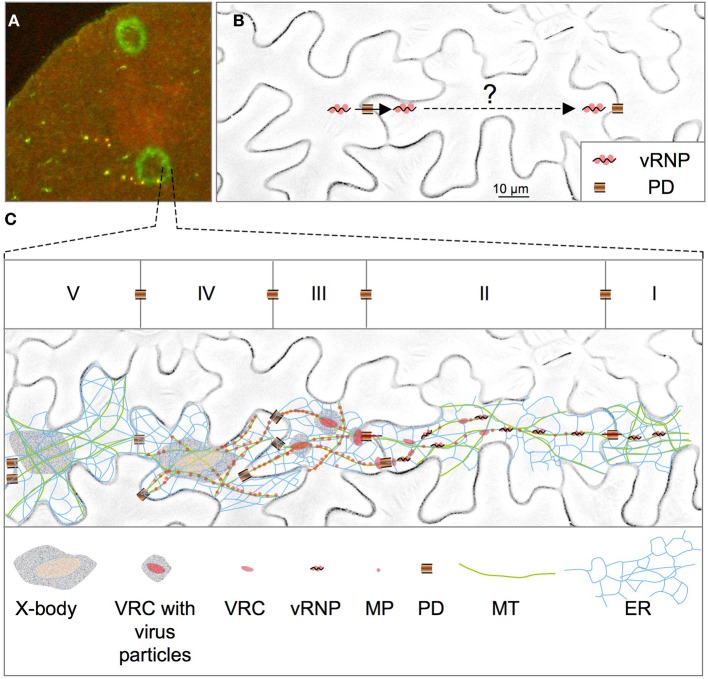
**Spreading of TMV infection. (A)** Section of a *Nicotiana benthamiana* leaf showing infection sites of TMV-MP:GFP at 5 days post-infection. The outer rim of the infection site represents the leading front of the spreading virus. The infection site appears as a fluorescent ring because MP:GFP accumulates only transiently and is then degraded. **(B)** Upon entering a cell through plasmodesmata (PD) the vRNP must be replicated and transported across the diameter of the cell before it can infect the next cell. *N. benthamiana* epidermal cells are shown. **(C)** Current model for TMV movement and the transient activities of the MP during this process. The model is projected on the pattern of five epidermal cells (depicted as I–V) at the leading front of infection. Infection of a cell (II) starts with the association of the vRNP with sites on the ER that concur with microtubules (MT) and allow the establishment of new viral replication complexes (VRCs). Following initial vRNA replication, some VRCs or VRC subcomplexes (containing vRNPs) are detached from their anchorage sites and transported via the ER through PD to infect a new cell (I). As infection already spreads, other initial VRCs continue translation and replication and increase in size over time and finally produce virus progeny (III–V). MP produced in cells behind the leading front accumulates in the MP-producing VRCs and subsequently along MT before degradation (III–IV). At late stages of infection, VRCs cease to produce MP but may continue replication to produce virion particles (IV). In a final stage all MP has disappeared (except from PD where MP is stable) and cells contain “X-bodies” with surrounding virion particles (V).

**Figure 2:**
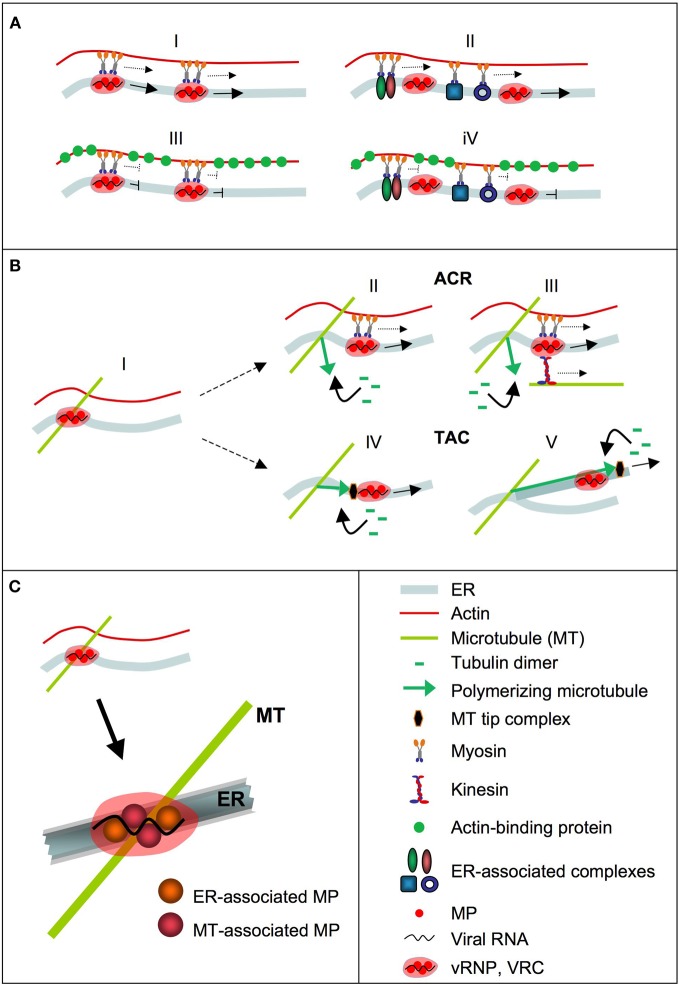
**Mechanisms in ER-mediated vRNA transport.** vRNA is depicted in the form of a VRC. **(A)** Role of the ER-associated actin cytoskeleton in VRC transport. ER-associated VRCs may represent cargo for myosins that transport the VRCs along the ER in an actin-dependent manner (I). Transport of VRCs along the ER may also be facilitated by myosin-driven macromolecular mass flow along the membrane (II). Overexpression of actin-binding protein interferes with myosin movements along actin filaments and therefore with myosin-supported VRC transport along the membrane (III and IV). Disruption of actin interferes with the ability of myosins to facilitate directional transport but does not completely abolish VRC transport along the membrane (not shown). **(B)** Role of microtubules (MT) in ER-associated VRC transport. VRCs attached at ER/MT intersections (I). In the Attachment Complex Release (ACR) mechanism, locally induced MT polymerization releases the VRCs from attachment sites for ER-guided transport (I) with support by myosins (II), kinesins, or both (III). In the Tip-Attachment Complex (TAC) mechanism, locally induced MT polymerization causes VRC detachment and also provides the force to accelerate VRC movement along the ER, ether by pushing the VRC along existing membrane tubules (IV), or by extending ER tubules carrying the VCR (V). **(C)** vRNPs (VRCs) may represent higher order structures in which MP molecules with different folds that expose domains for interaction with the ER or MT, respectively, are combined with vRNA and other viral and host factors.

To facilitate the cell-to-cell spread of this large vRNP, the MP increases the size exclusion limit of plasmodesmata (PD) (Wolf et al., 1989; Oparka et al., 1997), the intercellular communication channels in the plant cell walls (Heinlein, 2002; Heinlein and Epel, 2004; Maule, 2008). Although the virus may spread into adjacent cells from replication sites established near the channels (Szécsi et al., 1999; Kotlizky et al., 2001), the spread of infection through whole tissues depends on the transport of the virus across the diameter of the cells (Figure 1). Thus, from PD used for cellular entry the vRNP first binds to intracellular sites to establish new viral replication complexes (VRC) and to amplify vRNA. And, in a second step, the newly replicated vRNPs are transported from VRCs to the PD that link the infected cell to neighboring, yet non-infected cells. One important mechanism that may contribute to virus transport is cytosplasmic streaming (Verchot-Lubicz and Goldstein, 2010). Despite the large size of the vRNP, this process may contribute to distributing the vRNPs around in the newly entered cells before they attach to new sites for replication. However, once replicated, the new vRNP particles must be specifically targeted from the replication sites to PD, which likely depends on more specialized mechanisms. Consistent with this potential requirement for transport to PD, numerous studies highlighted the importance of MP associations with dynamic endoplasmic reticulum (ER) membranes and components of the cytoskeleton (Heinlein et al., 1995, 1998; Boyko et al., 2000a, 2007; Brandner et al., 2008; Sambade et al., 2008) (for review, see Niehl and Heinlein, 2011; Peña et al., 2012). According to the current model (Figure 1), infection of a new cell starts with the association of the vRNP with sites on the ER. Translation and replication of the vRNA leads to the formation of distinct, ER-localized VRCs that increase in size over time and finally form so-called X-bodies that produce virus progeny. Just following infection of the new cell and before the VRCs increase in size, the virus already moves further into adjacent cells, presumably in the form vRNP- containing VRCs or VRC sub-complexes that detach from their ER anchorage sites for subsequent transport via lateral diffusion along the ER membrane. Since the ER network is contiguous between cells through PD (Maule, 2008) it provides a direct pathway for guiding the vRNP/VRC from replication sites into adjacent cells. Consistent with transport along the ER, the MP has predicted transmembrane domains essential for membrane association and virus movement (Fujiki et al., 2006). Transport along the ER is facilitated by the ER-associated actin system and can be blocked by overexpression of actin-binding protein (Hofmann et al., 2009) (Figure 2A). The same conditions also blocked the myosin-dependent transport of Golgi complexes thus indicating a role of myosin motors in vRNP/VRC trafficking. These motors may act in vRNP/VRC transport through binding the vRNP/VRC as a transport cargo, or indirectly, by supporting bulk flow macromolecular trafficking in the membrane (Figure 2A) (Hofmann et al., 2009). However, at present it remains unclear whether vRNP/VRC transport along the ER indeed depends on the ER-associated actin system or whether the ER alone could provide sufficient membrane-associated motility for virus movement. The latter proposal is suggested by reports showing that ER membranes maintain lateral macromolecular transport upon actin disruption or myosin inhibition, albeit with reduced efficiency (Runions et al., 2006; Griffing, 2010). Although the disruption of actin should reduce the efficiency of vRNP particle/VRC transport along the membrane, the intercellular spread of infection was not immediately inhibited upon actin disruption for 24 h, as was seen by the uninterrupted expansion of infection sites in leaves under these conditions (Hofmann et al., 2009). However, this observation may be expected given that the movement of only few genome units is sufficient for the spread of infection (Li and Roossinck, 2004; Gutierrez et al., 2010). Nevertheless, inhibition of virus movement at the level of infection sites was observed upon long-term inhibition of the actin-myosin system (≥3 days), either by exposure to actin or myosin antagonists, or by silencing actin or myosin expression (Kawakami et al., 2004; Liu et al., 2005; Harries et al., 2009a,b). Yet, in this case, it remains to be seen whether this result implies a direct role of actin and myosin in VRC transport along the ER membrane or whether this observation could also be explained by indirect effects, for example by compromising the role of the actin cytoskeleton in maintaining the dynamic structural integrity of the ER (Wright et al., 2007; Hofmann et al., 2009; Sparkes et al., 2009b). It is also yet unclear whether the MP itself interacts with actin or myosin to facilitate movement. A direct interaction was suggested by early studies in fixed protoplasts showing an alignment of antibody-labeled MP to phalloidin-rhodamine labeled actin filaments (McLean et al., 1995). However, recent *in vivo* studies in leaves argued against this observation (Hofmann et al., 2009). An interaction of MP with actin was then recently again suggested by the finding that MP causes actin severing *in vitro* and that the stabilization of actin filaments with phalloidin inhibits the ability of MP to increase the SEL of PD *in vivo* (Su et al., 2010). Although these observations provide compelling evidence for a role of actin severing in the regulation of the PD SEL, it still remains uncertain whether MP interacts with actin *in vivo* and whether its actin severing activity would occur at PD. Recent studies indicate that the virus and its VRCs interact with actin filaments via replicase and that both replicase and actin filaments play a role in VRC formation and growth (Liu et al., 2005). Possibly, interactions between replicase and actin filaments play a role also in supporting the efficiency of vRNP/VRC movement along the ER.

## vRNA transport is controlled by the microtubule system

While further studies are needed to decipher the interactions of MP with the actin cytoskeleton, there is compelling evidence for the binding of MP to microtubules, tubulin, and microtubule-associated factors (Heinlein et al., 1995; McLean et al., 1995; Padgett et al., 1996; Heinlein et al., 1998; Boyko et al., 2000a,b; Kragler et al., 2003; Ashby et al., 2006; Ferralli et al., 2006; Brandner et al., 2008; Sambade et al., 2008). MP binding to microtubules stabilizes them against disruption by polymerization inhibitors, salt, and cold (Boyko et al., 2000a; Ashby et al., 2006; Ferralli et al., 2006). The MP also has the capacity to induce changes in the microtubule array through interaction with microtubule nucleation complexes (Ferralli et al., 2006), a finding supported by the ability of MP to interact with microtubule assembly factors, such as GFP-fused EB1 (Brandner et al., 2008) and γ-tubulin (Sambade et al., 2008). *In vivo* functional studies correlated the interaction between MP and microtubules with MP function in virus movement (Boyko et al., 2000a,c, 2007). However, it is still unclear whether the interaction of MP with microtubules is a requirement or rather a consequence of function. Nevertheless, with respect to interactions of MP with microtubules it is important to differentiate between early and late stages of infection. During early stages of infection, thus in cells at the front of spreading infection sites in leaves, the protein interacts with microtubules in the form of ER-associated mobile particles, whereas at later stages, thus in cells behind the front, the protein accumulates along the filaments (Boyko et al., 2007; Sambade et al., 2008). The latter feature occurs in cells behind the spreading front of the virus and is thus dispensable for virus movement. Accumulation along microtubules interferes with MP particle transport (Boyko et al., 2007) and may play an important role in halting the spread of vRNPs/VRCs as soon as the virus moved into new cells. This negative regulation of MP activity involves the microtubule-associated protein MPB2C that enhances the sequestration of MP by microtubules and may represent a central regulator in intercellular macromolecular trafficking (Kragler et al., 2003; Curin et al., 2007; Winter et al., 2007). Consistent with the notion that the movement process itself is independent of microtubule-aligned MP, infection spreads with similar efficiency if the amount of MP produced during infection is reduced and microtubule-aligned MP is only rarely observed (Heinlein et al., 1998).

## Analysis of MP activity in RNA transport in the absence of infection

To study MP activity in RNA transport, several studies employed ectopic expression since the protein maintains function under these conditions. Thus, ectopically expressed MP complements for movement of MP-deficient virus and supports the movement of other viruses (Deom et al., 1987; Meshi et al., 1987; Holt and Beachy, 1991; Cooper et al., 1995, 1996; Atabekov et al., 1999; Vogler et al., 2008). When expressed ectopically, the MP also maintains the association with cellular targets such as the ER, microtubules, and PD, even in the absence of infection (Heinlein et al., 1998; Sambade et al., 2008; Boutant et al., 2010). Interestingly, ectopically expressed MP also maintains the capacity to form mobile complexes that strongly resemble in shape, localization and motility behavior the mobile MP complexes (which may represent early VRCs) seen at the leading front of infection (Boyko et al., 2007; Sambade et al., 2008). The mobile MP complexes formed by transiently expressed MP in the absence of infection are functionally relevant since their formation is affected by conditional amino acid mutations in MP that also affect the formation of the mobile MP complexes/early VRCs and virus movement during infection in a temperature-sensitive manner (Boyko et al., 2007; Sambade et al., 2008). It appears remarkable that ectopically expressed MP forms particles that strongly resemble those formed during infection, despite that no vRNA is present. This may indicate that MP and vRNA join existing mechanisms for RNA transport during infection. The movements of the MP complexes are directional and occur in a stop-and-go manner along the ER and always also in contact with underlying microtubules (Sambade et al., 2008). Pausing of the movements occurs at microtubule sites that may act as anchorage sites at the ER and at which the mobile complexes may be assembled and controlled. In the context of infection, these sites may represent sites for attachment of VRCs that may subsequently detach again for movement, or may remain attached and grow into larger virion-producing VRCs (X-bodies). The observation of halting movements at microtubule sites is consistent with other reports indicating that cortical plant microtubules and underlying ER tubule junctions play a role in guiding and controlling the movements of large complexes, including RNA-containing complexes and organelles, in the cortical cytoplasm (Crowell et al., 2009; Gutierrez et al., 2009; Cai and Cresti, 2012; Hamada et al., 2012). According to the roles of the actomyosin system in supporting directional macromolecular transport along the ER (Sparkes et al., 2009a,b), the movements of the transiently expressed MP complexes along the ER are reduced in the presence of actin inhibitors (Sambade et al., 2008). Interestingly, upon application of microtubule polymerization inhibitors the complexes remained stably anchored at microtubule sites (Sambade et al., 2008). Given that MP was shown to interact with GFP-fused microtubule-tip protein EB1 and with the microtubule-organizing center component γ-tubulin, this may suggest that the movements of the MP complexes and early VRCs depend on induced microtubule polymerization (Sambade et al., 2008). A role of microtubule polymerization in TMV movement indeed appears likely since it was shown that herbicide-resistant tobacco mutants that are affected in microtubule polymerization dynamics are also compromised in their ability to support efficient virus movement (Ouko et al., 2010). We proposed two mechanisms by which microtubule polymerization could support virus movement (Sambade and Heinlein, 2009) (Figure 2B). Whereas the Attachment Complex Release (ACR) mechanism uses microtubule polymerization to release the vRNPs/VRCs from attachment/assembly sites, the Tip-Attachment Complex (TAC) mechanism uses microtubule polymerization, thus growing microtubules, to push the complexes along the ER. The TAC mechanism plays an important role in ER tubule motility (Waterman-Storer and Salmon, 1998) and may indeed provide strong pushing forces as, for example, shown by the ability of polymerizing microtubules to move whole nuclei through the cytoplasm (Zhao et al., 2012).

## Analysis of MP activity in RNA transport at the level of labeled RNA

**Figure 3:**
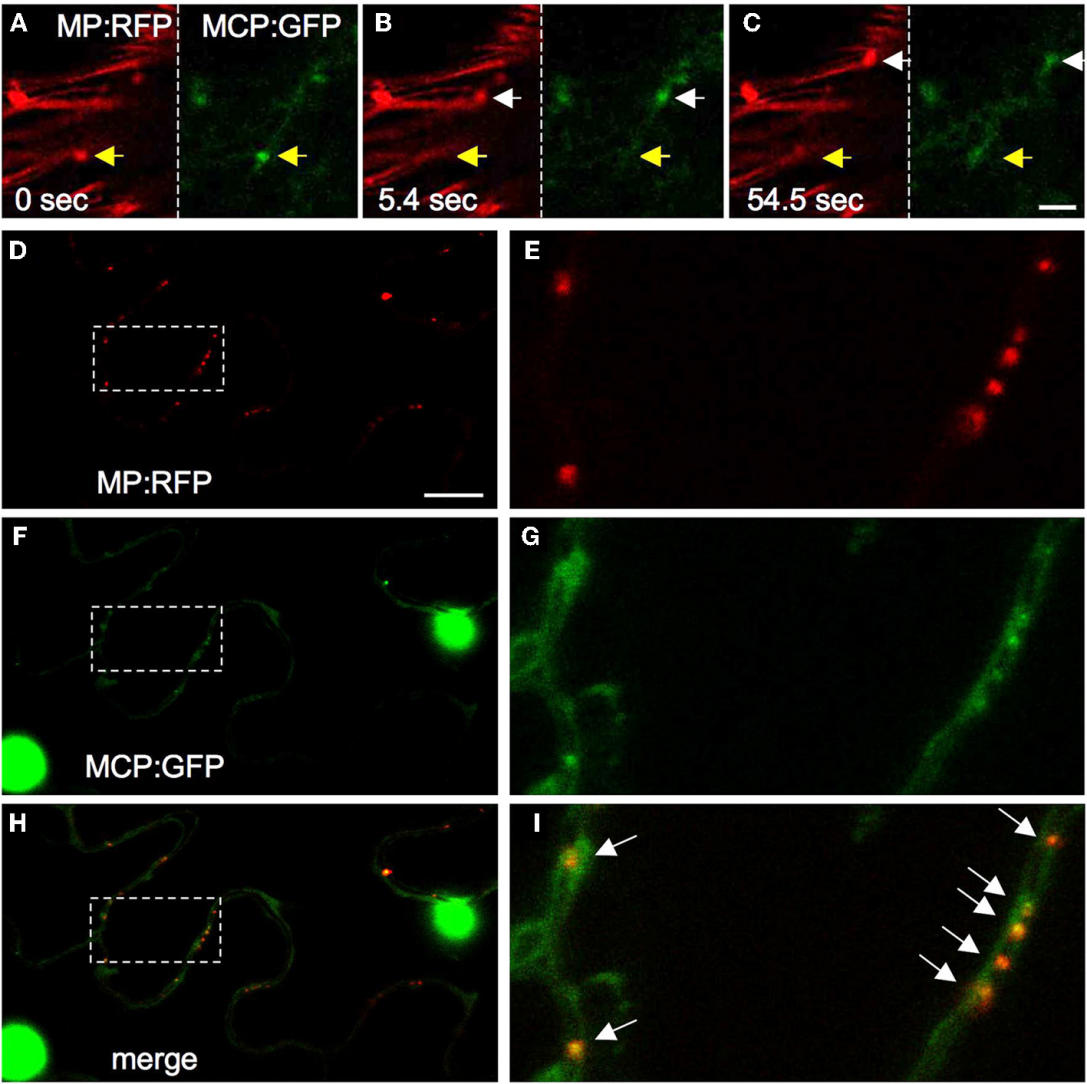
**Detection of MP mRNA with MCP. (A–C)** Examples of video frames showing the coinciding movements of MP:RFP (red) and MP:RFP mRNA (green, labeled with MCP:GFP) particles. Size bar, 5 μm. **(D–I)** MP:RFP (**D** and **E**, red) and MP:RFP mRNA (**F** and **G**, green, labeled with MCP:GFP) coincide at the cell wall, presumably in PD (arrows in **I**). **H** and **I** are merged images of **D** and **F**, and **E** and **G**, respectively. **E**, **G**, and **I** show enlargements of the area highlighted in **D**, **F**, and **H**. Size bar, 10 μm.

As collected evidence provides indications that the *in vivo* observed mobile ER-associated MP-containing complexes serve as the vehicle to transport vRNA into non-infected cells (Boyko et al., 2007; Sambade et al., 2008), it is important to determine that the particles indeed contain vRNA. Several methods for the *in vivo* labeling of specific RNA molecules are available and have been recently reviewed (Christensen et al., 2010). Earlier studies employed in situ hybridization of biochemically fixed, TMV-infected tobacco BY-2 protoplasts with digoxigenin-labeled probe to show in separate co-staining experiments that vRNA colocalizes with MP:GFP, with immunolabeled replicase, with immunolabeled ER, and with immunolabeled microtubules. The overlapping patterns observed are consistent with the presence of vRNA in small and large VRCs, with vRNA/VRCs present in particles along microtubules, and with a role of microtubules in the cellular distribution of vRNA/VRCs (Más and Beachy, 1999, 2000). More recently, microinjection of Cy3 pre-labeled vRNA into tobacco trichome cells was used to demonstrate that the microinjected vRNA immediately forms granules, associates with the ER in a 5′CAP-dependent manner, and moves along the ER/actin network (Christensen et al., 2009). The directional movement pattern of the labeled vRNA granules in trichomes may resemble the pattern of MP particle movements in cells at the virus front in leaves. Nevertheless, it appears probable that the vRNA granules observed in these microinjection experiments differ in nature and/or composition from mobile MP particles seen upon ectopic expression of tagged MP (Sambade et al., 2008) and from MP particles observed in cells at the spreading virus front in leaves (Boyko et al., 2007). Unlike the movements of the particles tagged with transiently expressed MP, the movement of the microinjected vRNA was insensitive to treatments with microtubule polymerization inhibitors. Moreover, microinjected vRNA was incompetent for movement between cells even in transgenic, MP-expressing plants (Christensen et al., 2009). Previous evidence indicated that MP-mediated macromolecular transport requires physical association of the transported molecules with MP (Waigmann and Zambryski, 1995). Thus, although vRNA molecules move between cells if co-injected with MP (Nguyen et al., 1996), microinjected RNA may not find plant-expressed MP and thus fails to form movement-competent complexes. It is also possible that microinjected RNA preferably associates with factors for translation and replication rather than with MP. Another approach to localize vRNA *in vivo* involves the use of Pumilio, an RNA binding protein, coupled to bimolecular fluorescence complementation (BiFC) (Tilsner et al., 2009). Upon introduction into infected *N. benthamiana* cells this system revealed the localization of vRNA in large VRCs and in smaller discrete particles throughout the peripheral cytoplasm. The Pumilio detection system is less invasive than microinjection. However, its application appears to be associated with a certain level of background caused by unspecific BiFC and promiscuity of Pumilio binding to RNA (Tilsner et al., 2009). In our own efforts, we have applied the non-invasive RNA detection system based on the MS2 phage CP (Bertrand et al., 1998). Expression of this protein fused to a reporter such as GFP allows specific detection of RNA molecules carrying stem-loop sequences derived from the origin of assembly (OAS) of the phage. To reduce the background of MS2-CP:GFP (MCP), the protein is fused to nuclear localization signal (NLS) so that the protein localizes to the nucleus and is retained in the cytoplasm only if associated with the RNA binding target. Our attempts to localize vRNA were not successful so far since the insertion of the MS2 OAS sequences into the vRNA interferes with TMV infectivity. However, we successfully used this system to detect the mRNA of ectopically expressed MP (Sambade et al., 2008). As described above, ectopically expressed MP:GFP is functional and forms mobile, ER-associated particles that are functionally related to the MP particles expressed in cells at the leading front of spreading infection sites in leaves. Co-expression of MP:RFP with MCP allowed the detection of mobile MP:RFP mRNA signal that coincides with mobile MP:RFP particles (Figures 3A–C). Detection of the mRNA is highly specific and depends on the presence of the OAS stem-loops in the mRNA sequence (Sambade et al., 2008). These observations revealed that the mobile complexes formed by transiently expressed MP on the ER contain MP mRNA. Moreover, MP:RFP mRNA was detected at PD (Figures 3D–I) in a manner dependent on MP (Sambade et al., 2008). However, because of the fast tracking motions and the low level of MS2-CP:GFP labeling the further analysis of the mobile RNA complexes is difficult. Increasing MCP expression to improve signal strength causes considerable background labeling that can strongly interfere with the interpretation of the RNA labeling results. While the MS2 system is further developed (Wu et al., 2012), our current efforts concentrate on testing the application of other RNA binding protein-dependent RNA-labeling systems, such as λN (Daigle and Ellenberg, 2007). New approaches also include the development of sequence-specific RNA detection using aptamer-binding dyes (Lux et al., 2012). We hope that these new attempts will allow us provide further information about the composition of the MP-tagged mRNA particles and the pathway that guides them to PD.

## Outlook

Despite important insights into the interactions of MP with host cell components have been achieved we are still far away from understanding the molecular mechanism by which this protein facilitates the transport of vRNA between cells. More efforts are needed to reliably detect RNA *in vivo* and to study cell-to-cell transport at the RNA level. Further studies will also have to focus on the analysis of the composition of the MP particles. The MP particles seen upon transient expression may differ in composition from those formed at the leading front of infection as there is evidence that the viral replicase contributes to virus movement (Hirashima and Watanabe, 2001; Guenoune-Gelbart et al., 2008) and that the virus moves as a VRC (Kawakami et al., 2004). However, identifying the composition of the particles formed by transiently expressed MP may provide direct clues of how the MP interacts with the existing RNA transport pathway. It will be important also to determine if MP-mediated vRNA transport requires the activity of motor proteins. Although evidence for the involvement of myosin in virus movement has been provided (Harries et al., 2009a) it is unclear where exactly in the cell myosin activity is involved. Myosin motor activity accelerates macromolecular transport along the ER membrane (Sparkes et al., 2009b) and may also accelerate the diffusion of vRNPs/VRCs along the membrane towards and through PD. It will be important to determine whether MP or the replicase could represent cargo for specific myosins or whether myosins rather interact with other ER-localized complexes and support virus movement indirectly through macromolecular bulk flow in the membrane.

Another question concerns the range of molecules that the MP moves cell-to-cell. MPs can support the trafficking of different viruses and certain recombinant MPs, including the MP of TMV, were shown to facilitate intercellular trafficking of different viral nucleic acids prepared *in vitro* and co-injected into a cell, both of which is consistent with their sequence non-specific nucleic acid binding activity (Ding, 1998; Heinlein and Epel, 2004). However, although sequence non-specific binding of nucleic acids may allow MP to facilitate the transport of any bound RNA, movement of VRC subcomplexes or of whole VRCs likely increases specificity for the transport of vRNAs, which are produced in VRCs. Thus, MP-mediated transport of RNAs other than vRNA may represent an exception. Nevertheless, the observation that MP enhances the intercellular spread of induced GFP-transgene silencing (Vogler et al., 2008) may indicate that the protein facilitates the movement of small RNA molecules. Spreading of virus and/or host-derived small RNAs at the leading front of infection by MP may induce transient changes in target gene expression and in the susceptibility of the cells for the moving virus (Amari et al., 2012). Understanding vRNA transport and virus movement may be enhanced by including the analysis of small RNA activity at and behind the virus front.

Further important insights into MP functions will also depend on determining the structure of MP. The protein may assume different structures *in vivo* since the protein domain involved in interactions with microtubules overlaps with predicted transmembrane domains involved in the association with membranes (Boyko et al., 2000a; Brill et al., 2004; Fujiki et al., 2006). The protein acts a dimer or multimer (Brill et al., 2004; Boutant et al., 2010) and thus may form higher-order structures that allow simultaneous exposure of different interaction domains through combining MP molecules with specific folds (Figure 2C). Since the protein is phosphorylated and ubiquitinylated *in vivo* (Lee and Lucas, 2001; Ashby et al., 2006) further studies may identify the roles of these post-translational modifications in directing the folding and function, and subsequent turnover of the protein.

It will also be important to determine how far the observations described for TMV and summarized herein also apply to the movement of other RNA viruses. Mechanisms involved in the cell-to-cell movement of viruses have been reviewed in several recent overview articles (Lucas, 2006; Harries and Ding, 2011; Niehl and Heinlein, 2011; Schoelz et al., 2011; Ueki and Citovsky, 2011) and show a role of membranes and the cytoskeleton as a common scheme. However, important differences certainly do exist. For example, viruses that move between cells in the form of virions must depend on molecular mechanisms other than viruses that move in a non-encapsidated form like TMV. Moreover, some viruses induce tubular transport structures inside PD, which involves the displacement of the ER from the PD channel. Thus, unlike TMV these viruses cannot rely on the ER as a structure that guides them to the pore. Here, endocytic vesicle trafficking can play a role, as indicated for nepoviruses (Amari et al., 2010). Whereas the actin cytoskeleton seems to play direct or indirect roles in the movement of many viruses there is yet limited evidence for microtubules playing a general role in virus movement. A recent study demonstrated that the triple gene block protein 1 (TGBp1) of *Potato mop-to virus* (PMTV) interacts with microtubules (Wright et al., 2010). The accumulation of this MP along microtubules is observed only in cells behind the infection front and the treatment of the plants with microtubule-disrupting agents does not interfere with further lesion growth. Although this shows that a wholly intact microtubule cytoskeleton is not required for virus movement, the TGB1 may still interact with the microtubule system in the presence of inhibitors and form microtubule-interacting structures also in cells at the virus front as was shown for TMV (Seemanpillai et al., 2006; Boyko et al., 2007). The detection of mobile RNA particles in cells at the virus front and evidence supporting a functional role of these particles in virus cell-to-cell movement is an achievement so far unique for TMV. Certainly, further studies are needed to reveal whether these observations reflect a general mechanism also applying to other viruses and RNA transport processes in plants.

However, a role of RNA-enriched mobile particles in the transport of RNA viruses and other RNA molecules is certainly consistent with similar observations in other systems. These include neuronal granules for transport of mRNAs along axons and dendrites for localized translation at synapses (Kiebler and Bassell, 2006; Doyle and Kiebler, 2011), RNA granules in Drosophila polar development (Ferrandon et al., 1997; Hachet and Ephrussi, 2004) and RNA transport particles in budding yeast (Lange et al., 2008). The key principles of mRNA localization mechanisms in these systems have been established and many players identified (Palacios et al., 2001; Holt and Bullock, 2009; Martin and Ephrussi, 2009; Shahbabian and Chartrand, 2012). Thus, RNA transport particles can contain several or only one mRNA (Besse et al., 2009; Macdonald, 2011; Batish et al., 2012) and their transport is usually determined by cis-acting localization elements in the 3′UTR of the mRNAs. These elements are recognized by specific families of trans-acting RNA-binding proteins and the set of proteins that binds to the mRNA already during and after transcription in the nucleus plays a role in determining the ultimate location and fate of the mRNA within the cytoplasm. An important concept is that the mRNAs are translationally repressed during transport, and recent studies have suggested a role of non-coding RNAs and miRNAs in the transport particles (Besse and Ephrussi, 2008). The particles are transported by motor proteins (myosins, kinesins, and dyneins) along microtubules and microfilaments and their anchorage at their final destinations depends in many cases on actin. Collectively, the targeted delivery of RNA molecules in the form of particles, the presence of trans-acting proteins binding to cis-acting RNA elements, and the role of the cytoskeleton are characteristics reminiscent of those of the observed MP particles, i.e., their RNA content, the ability of MP to bind RNA, translational inhibition during transport (Karpova et al., 1997, 1999), and the role of the cytoskeleton. However, whether the composition of the RNA particles formed or used by MP and the mechanism that transports them along the ER have indeed any similarity with the RNA particles in other systems remains to be seen.

### Conflict of interest statement

The authors declare that the research was conducted in the absence of any commercial or financial relationships that could be construed as a potential conflict of interest.
